# Optical coherence tomography angiography of the peripapillary capillaries in primary open-angle and normal-tension glaucoma

**DOI:** 10.1371/journal.pone.0184301

**Published:** 2017-09-15

**Authors:** Ryoko Igarashi, Shun Ochiai, Yuta Sakaue, Aki Suetake, Ryu Iikawa, Testuya Togano, Fuuko Miyamoto, Daiki Miyamoto, Takeo Fukuchi

**Affiliations:** Division of Ophthalmology and Visual Science, Graduate School of Medical and Dental Science, Niigata University, Niigata, Japan; Bascom Palmer Eye Institute, UNITED STATES

## Abstract

**Objectives:**

To evaluate the vascular architecture of the radial peripapillary capillaries (RPCs) and its relation with visual function in patients with open-angle glaucoma (OAG) and normal-tension glaucoma using spectral-domain optical coherence tomography (SD-OCT) angiography.

**Subjects and methods:**

Clear OCT angiography images of blood vessels in the optic disc and peripapillary retina were obtained from 52 patients (52 eyes) aged 55.42±10.64 (range 28–72) years with primary OAG. The mean spherical equivalent was -3.19±2.31 diopters, and the mean deviation (MD) of the central 24/30-2 threshold test using the Humphrey Field Analyzer (HFA) was -10.47±7.99 dB. The correlations between the disappearance angle of the RPCs on OTC images, flow density (FD) and the circumpapillary retinal nerve fiber layer (cpRNFL) thickness, the angle of retinal nerve fiber layer (RNFL) defect, the MD and pattern standard deviation (PSD) values of the HFA central 24/30-2 threshold test using the Swedish interactive thresholding algorithm, the sensitivity threshold, age, corneal thickness, and refractive value were analyzed. In addition, the correlation between FD and the cpRNFL thickness was analyzed at FD measurement points.

**Results:**

FD was significantly correlated with cpRNFL thickness, PSD value, MD value, and sensitivity threshold, whereas the disappearance angle of the RPCs was significantly correlated with the angle of the RNFL defect (P<0.001), MD value (P<0.01), and sensitivity threshold (P<0.01). There was a negative correlation between FD and age (P<0.05). The Pearson correlation coefficient of FD and cpRNFL thickness in the area surrounding the optic disc revealed the most significant correlation in the inferior visual field (r = 0.851, P<0.001), followed by the superior visual field (r = 0.803, P<0.001) and then the temporal visual field (r = 0.653, P<0.001).

**Conclusion:**

SD-OCT angiography enabled thorough observation of the RPCs. FD and the disappearance angle of the RPCs were significantly and independently correlated with glaucoma-related functional and morphological changes in the optic nerve, suggesting that these two factors are novel functional and morphological indicators of visual defects due to glaucoma.

## Introduction

Spectrometer-based spectral-domain optical coherence tomography (SD-OCT) enables the rapid acquisition of many intraocular images, displaying the individual layers of the retina in detail, in addition to the entire morphology of the retina as in conventional OCT. SD-OCT is currently used frequently in clinical diagnosis and treatment [1–3], especially for glaucoma, because of its demonstrated utility in measuring the thickness of the peripapillary retinal nerve fiber layer (RNFL) and the retinal inner layers, including the ganglion cell layer, in the macular region. Furthermore, recent technological advances have led to the development of Doppler OCT and OCT angiography for the evaluation and measurement of intraocular blood vessels and blood flow. Doppler OCT measures the amount of blood flow using a special algorithm for computing the Doppler shift [4], while OCT angiography applies the principle of split-spectrum amplitude-decorrelation angiography (SSADA) to capture blood flow in the fundus as a moving object relative to the surrounding tissue as a static object, displaying the blood flow even in the capillaries of the retina [5,6].

Although glaucoma has been investigated intensely over the years, the elucidation of blood flow and microvasculature in the optic discs of patients with glaucoma remains a challenging research topic. Previous studies of glaucoma often reported fluorescein angiography findings of filling defects (no contrast enhancement) in the intrapapillary capillaries and the disappearance of radial peripapillary capillaries (RPCs) accompanied by an RNFL defect, generating discussions about whether these defects precede or follow visual field loss [7,8]. In addition, reduced intrapapillary, peripapillary, and macular blood flow was recently reported in patients with glaucoma, based mostly on laser speckle flowgraphy (LSFG) findings [9,10]. Using laser speckle flowgraphy, Shiga et al. also showed reduced blood flow in patients with pre-perimetric glaucoma, demonstrating the importance of blood flow measurement in the early stage of glaucoma [10]. Therefore, to establish criteria for glaucoma treatment, further studies are needed to elucidate the association between blood flow and structural changes.

In this study, we used OCT angiography to examine the peripapillary capillaries (PPCs) of patients with open-angle glaucoma (POAG and NTG patients), and investigated whether and how the disappearance angle of the RPCs and flow density (FD) were correlated with (1) morphological indicators: circumpapillary RNFL (cpRNFL) thickness and the angle of the RNFL defect; (2) functional indicators: sensitivity threshold and Humphrey Field Analyzer (HFA) 24/30-2 mean deviation (MD) and pattern standard deviation (PSD) values; and (3) clinical background factors: age, corneal thickness, and refractive value.

## Subjects and methods

### Patient inclusion

This study was approved by the Ethics Committee of Niigata University Medical and Dental Hospital and was conducted in accordance with the Declaration of Helsinki. We obtained written informed consent in advance from all participants or a legal guardian.

Between August 2014 and August 2016, Niigata University Medical and Dental Hospital conducted SD-OCT in 52 patients (24 men, 28 women) with POAG (aged 54.80±10.35 [range 29–69] years) or NTG (aged 55.81±10.96 [range 28–72]). Clear OCT angiography images of the optic disc were obtained from 53 eyes (30 right eyes, 22 left eyes). The mean spherical equivalent was -3.25±2.08 (-5.5 to 0) diopters (D) in POAG patients and -3.15±2.47 (-6.0 to +1.25) D in NTG patients, and the mean HFA 24/30-2 MD value was -12.61±7.60 (-24.47 to -1.41) dB in POAG patients and -7.24±14.65 (-28.61 to +0.8) dB in NTG patients. Among the 52 patients, 20 had (narrowly defined) POAG (20 eyes) and 32 had normal-tension glaucoma (32 eyes).

### Diagnosis of POAG and normal-tension glaucoma

Patients with glaucoma were recruited from Niigata University Medical and Dental Hospital in Niigata, Japan. All patients in the database had undergone a routine comprehensive ophthalmic examination that included assessment of best-corrected visual acuity using a 5-m Landolt chart, refraction, keratometry, slit-lamp examination, Goldmann applanation tonometry, gonioscopy, indirect ophthalmoscopy, dilated slit-lamp optic disc examination, central corneal thickness measurement (NSP-9900II; KONAN Medical, Hyogo, Japan), VF testing using the 30–2 Swedish Interactive Threshold Algorithm (SITA) Standard Strategy (HFA; Carl Zeiss Meditec, Dublin, CA, USA) and the 24–2 SITA Standard Strategy, and SD-OCT examination with the 3D-OCT 2000 (Topcon, Tokyo, Japan). Clinical diagnosis of glaucoma was based on Japan Glaucoma Society [3] guidelines. SITA was used to acquire reliable test results with <20% poor fixation and <15% false-positive and false-negative rates. Glaucomatous visual field loss was diagnosed based on Anderson and Patella’s criteria [11]. When patients had glaucoma in both eyes, the eye with visual loss was enrolled in this study. Exclusion criteria were refractive values with astigmatism > ±2D or spherical equivalent ≤ -6D, previous history of intraocular surgery, or eye disorders that adversely affect visual acuity or the visual field. Table 1 shows the clinical characteristics of the patients. There were no significant differences in age, sex, laterality, corrected visual acuity, sphere error, or visual field MD.

**Table 1:** Baseline characteristics of the study subjects.

Clinical characteristics	Total	POAG	NTG	P value
Age (years, range)	55.42±10.64 (28–72)	54.80±10.35 (29–69)	55.81±10.96 (28–72)	0.742
Sex (male/female)	24/28	12/8	12/20	0.118
Laterality (eyes)	30/22	13/17	17/15	0.406
Corrected visual acuity (log MAR)	0.05±0.09	0.07±0.05	0.02±0.15	0.152
Spherical error (diopter)	-3.19±2.31	-3.25±2.08	-3.15±2.47	0.874
Astigmatic error (diopter)	-0.60±0.59	-0.40±0.45	-0.68±0.62	0.105
Visual field MD (dB)	-10.47±7.99	-12.61±7.60	-7.24±14.65	0.129

The comparisons were performed using unpaired t-tests. Abbreviations: POAG, primary open-angle glaucoma; NTG, normal tension glaucoma; MD, mean deviation.

### Measurement and computation using the AngioVue system

**Fig 1:**
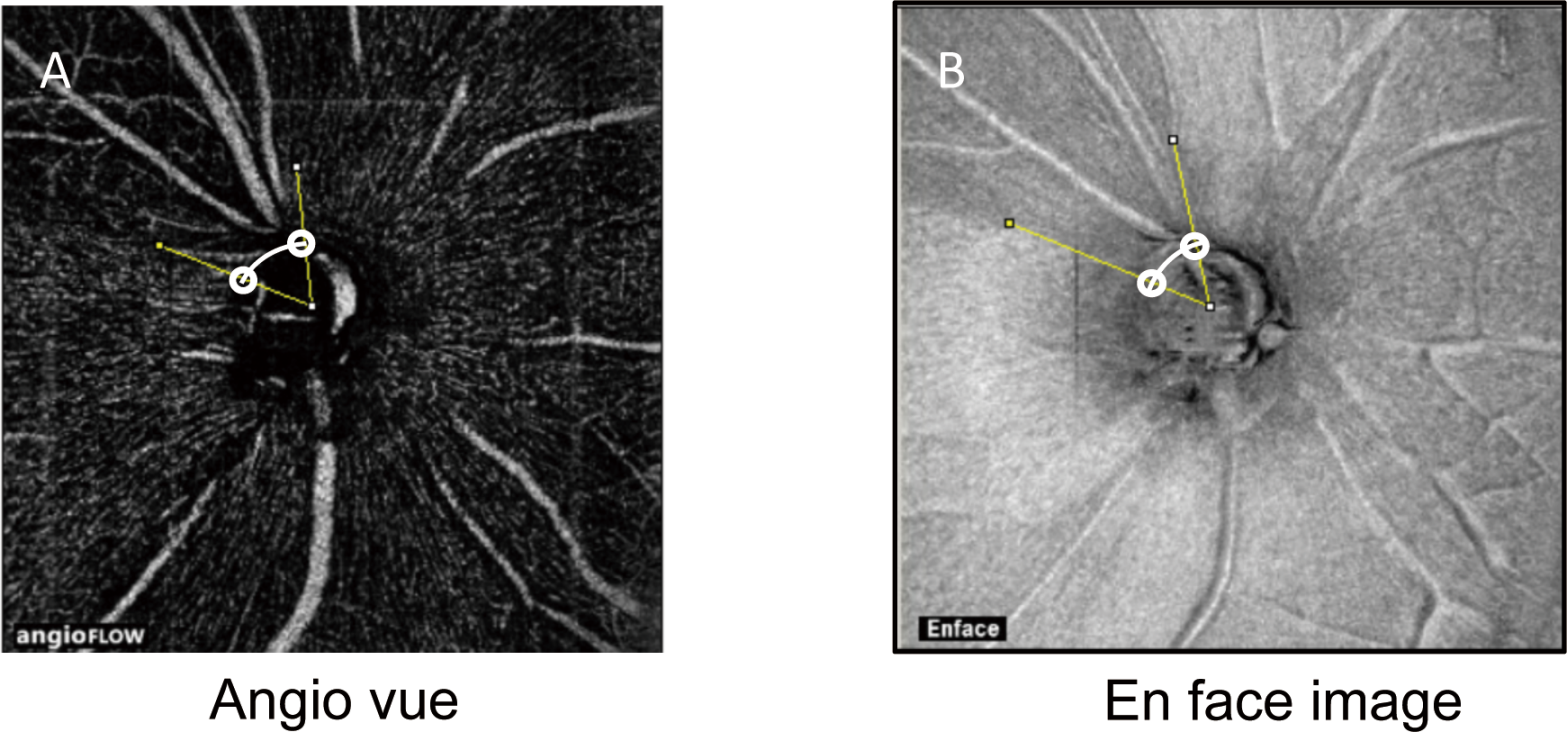
Method for determining the disappearance angle of the RPCs. The image processing and analysis software ImageJ was used to analyze OCT angiography images of the optic disc. At the edge of the optic disc, two sites where the RPCs disappeared and the RNFL defect was present were identified (white circles), and the center of the optic disc was connected to each site with straight lines (yellow lines). The angle generated by the two lines on OCT angiography images was defined as the disappearance angle of the RPCs (Fig 1A), and that on the en face images was defined as the angle of the RNFL defect (Fig 1B).

Using RTVue XR Avanti SD-OCT and Angio disc (Optovue, Fremont, CA, USA), OCT angiography was performed to generate images (4.5 mm × 4.5 mm) with the optic disc at the center (Fig 1A). Image exclusion criteria were a signal strength index (an indicator of image reliability) of ≤50 and unclear ocular vascular structures under visual inspection. On angioflow images, the RPCs and the segmentations were identified and examined, and the disappearance angle of the RPCs was measured. Of the four RPC images displayed on the monitor, the one containing a segment located 100 μm from the inner limiting membrane was used to measure the angle encompassing the RPCs. Segmentation error measured by OCTA and SD-OCT was digitally corrected.

### Measurement of cpRNFL thickness and division of the visual field

The corresponding en face image (the 6 mm × 6 mm image displaying the same segment located within 100 μm of the inner limiting membrane as in the above angioflow image) was then used to identify the area of the RNFL defect. The angle of the RNFL defect was measured using the same method as that used to determine the disappearance angle of the RPCs.

**Fig 2:**
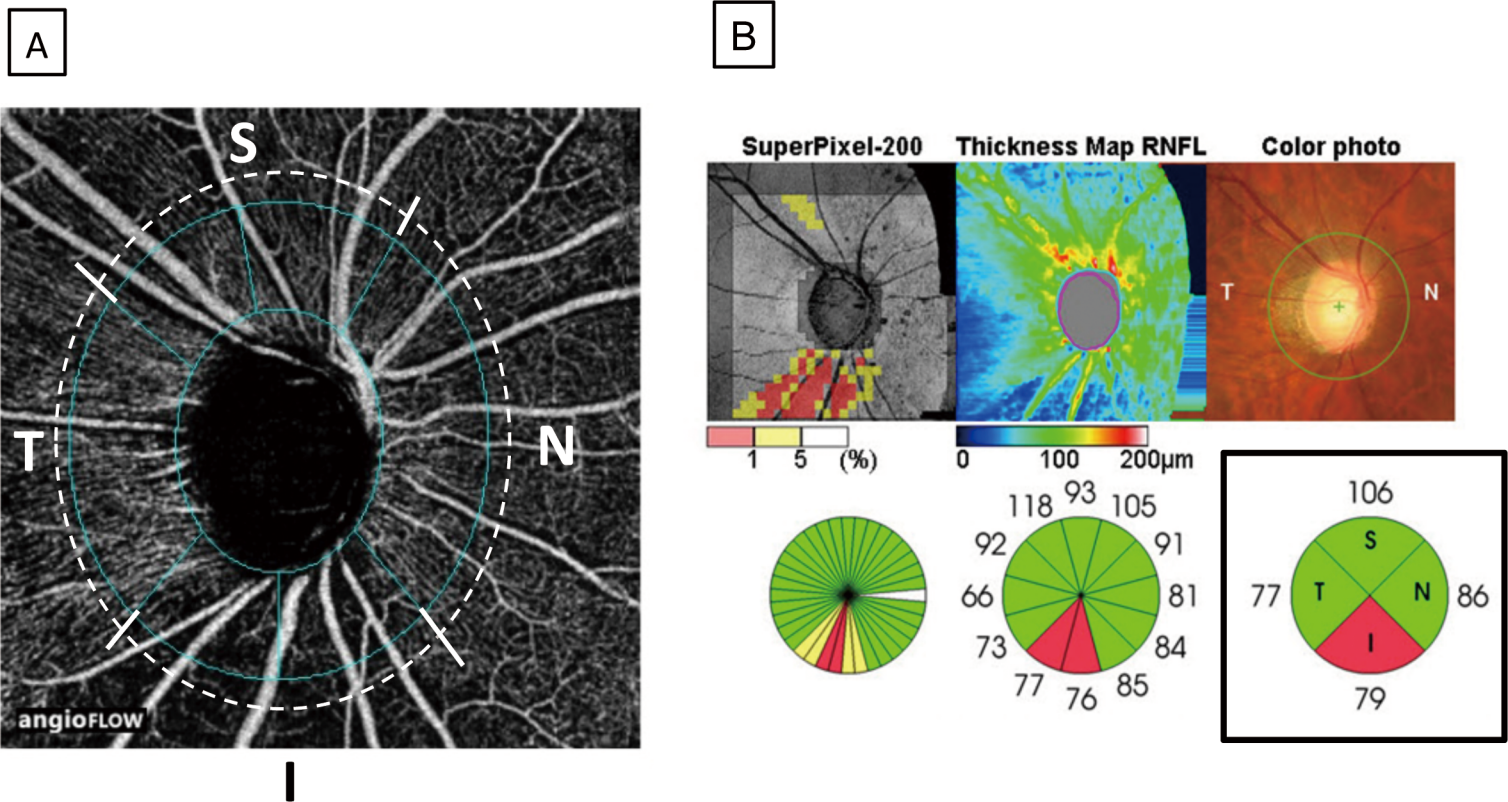
Method for dividing the peripapillary area into segments on OCT angiography and 3D-OCT images. Using the Angio disc mode of OCT angiography, the optic disc was divided into six areas (blue dotted lines), which were further grouped into four segments: a superior segment consisting of two superior areas (S), an inferior segment consisting of two inferior areas (I), a temporal segment (T), and a nasal segment (N) (Fig 2A). The four segments on OCT angiography images were matched with four segments of the optic disc that were automatically defined by the 3D-OCT optic disc map mode (Fig 2B, lower panel on the right).

Segmentation of the peripapillary area was performed based on cpRNFL thickness measured using the OCT angiography Angio Disc software (Fig 2A) and the 3D-OCT 2000 (Topcon) optic disc mapping function (Fig 2B). Exclusion criteria for 3D-OCT 2000 images were unclear optic discs or image quality ≤40.

### Statistical analysis

The angle encompassing the RPC area that disappeared (i.e., the disappearance angle of the RPCs) was measured using the Japanese version (1.48) of ImageJ, which was originally developed by the National Institutes of Health in the United States. On OCT angiography images, two sites at the edge of the optic disc where the RPCs disappeared were identified, and the center of the optic disc and each site were connected by a straight line. The angle generated by the two lines was defined as the disappearance angle of the RPCs (Fig 1A). When the RPCs disappeared at more than two sites, all of the sites were included in the measurement and were defined as the disappearance angle of the RPCs (Fig 1B). To ensure the reproducibility of the disappearance angle of the RPCs and the segmentation, the results of evaluations by three observers were averaged, and the intraclass correlation coefficient of measurement errors was analyzed using SPSS software version 21.0 (IBM Corp., Armonk, NY, USA).

Based on the above data, the Pearson correlation coefficient was used to analyze whether and how FD and the disappearance angle (DA) of the RPCs were correlated with the angle of the RNFL defect, cpRNFL thickness, PSD and MD values of the HFA 30/24-2 test, sensitivity threshold, age, reflection, and corneal thickness.

## Results

### Interobserver reproducibility of FD and DA measurements

The intraclass correlation coefficients calculated using SPSS software were used to determine the reproducibility and interobserver reliability of the disappearance angle of the RPCs and the angle of the RNFL defect measurements, as shown in Table 2. The intraclass correlation coefficient of the disappearance angle of the RPCs was r = 0.904 (P<0.001) in the overall population, r = 0.962 (P<0.001) in POAG patients, and r = 0.896 (P<0.001) in NTG patients. The intraclass correlation coefficient of the angle of the RNFL defect was r = 0.920 (P<0.001) in the overall population, r = 0.956 (P<0.001) in POAG patients, and r = 0.914 (P<0.001) in NTG patients.

**Table 2:** Reproducibility of the disappearance angle of the RPC and the RNFL defect. Intraclass correlation coefficients determined with SPSS.

	The disappearance angle of the RPCs		The angle of the RNFL defect	
	r	P value	r	P value
Total	0.904	<0.001	0.920	<0.001
POAG	0.962	<0.001	0.956	<0.001
NTG	0.896	<0.001	0.914	<0.001

Abbreviations: RPC, radial peripapillary capillaries; RNFL, peripapillary retinal nerve fiber layer; POAG, primary open-angle glaucoma; NTG, normal tension glaucoma.

### Correlations between different measurement parameters

**Fig 3:**
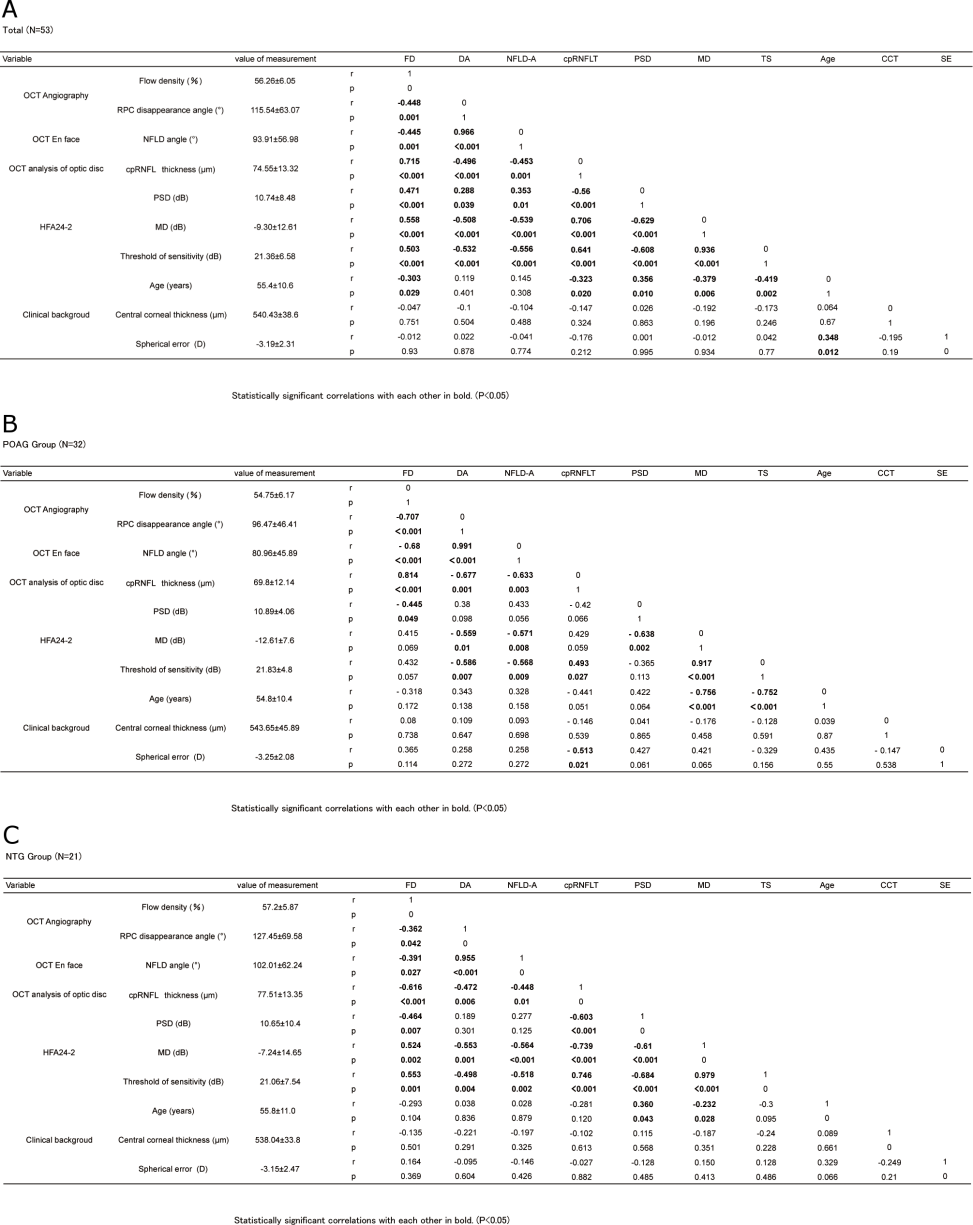
(a) Pearson correlation coefficient matrix for flow density, structural variables, visual field, and clinical background in subjects with glaucoma. (Total patients) (b) Pearson correlation coefficient matrix for flow density, structural variables, visual field, and clinical background in subjects with glaucoma. (POAG group) (c) Pearson correlation coefficient matrix for flow density, structural variables, visual field, and clinical background in subjects with glaucoma. (NTG group). Fig 3a-c shows the correlations between FD, the disappearance angle of the RPCs, and other parameters. Fig3a shows the correlations in the overall population, Fig 3b shows the correlations in the POAG patients, and Fig 3c shows the correlations in the NTG patients. Abbreviations: OCT, optical coherence tomography; HFA, Humphrey field analyzer; RPC, radial peripapillary capillary; NFLD, retinal nerve fiber layer defect; PSD, pattern standard deviation; MD, mean deviation; cpRNFLT, circumpapillary retinal nerve fiber layer thickness.

**Fig 4:**
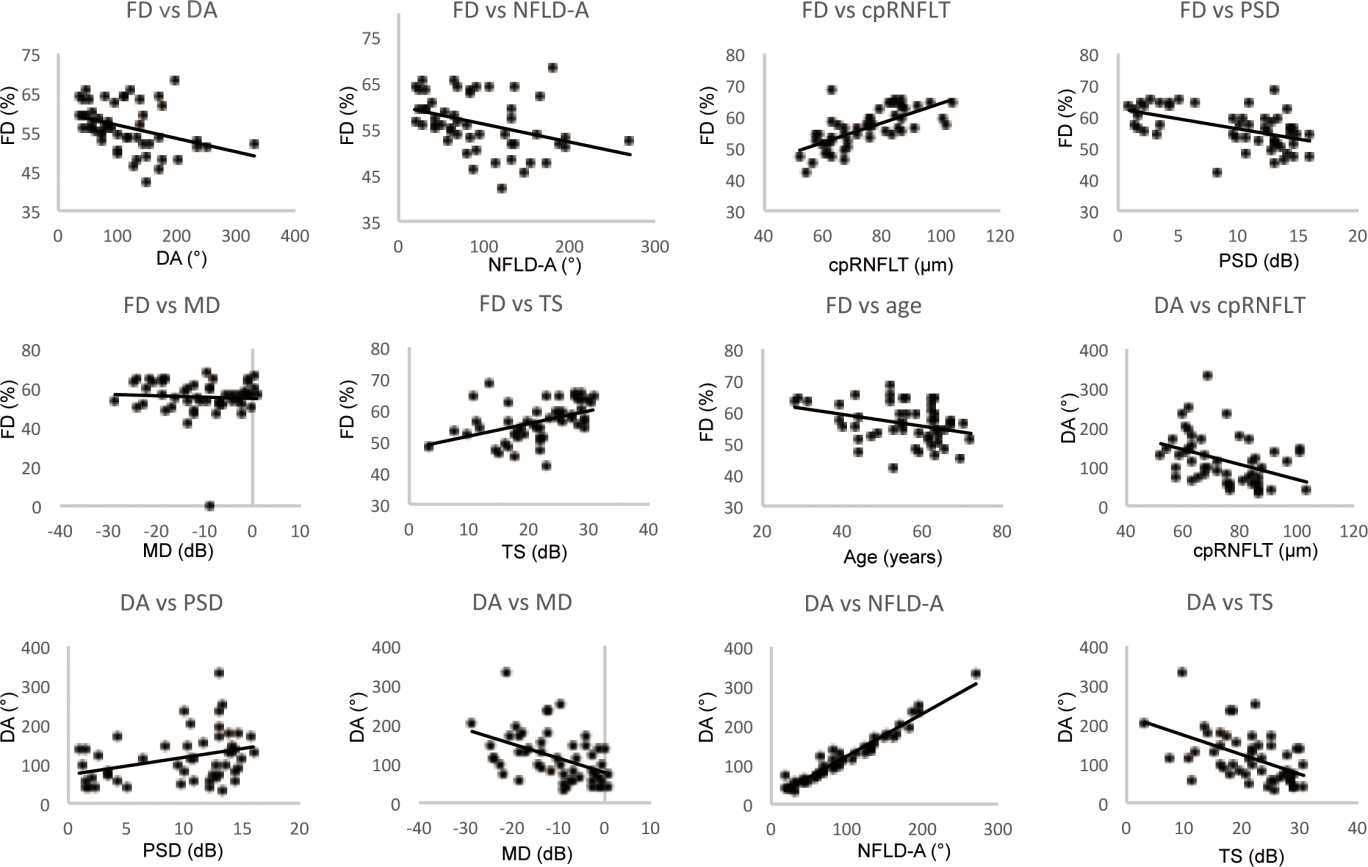
Scatterplots illustrating the linear associations between flow density (FD) on OCT angiography, the disappearance angle of the RPCs (DA), and other factors in glaucoma. The correlation between FD, DA, and other factors was analyzed, and measurement data are shown in Fig 3A–3C. A scatterplot of FD and DA (y-axis) and the retinal nerve fiber layer defect (NFLD) angle (NFLD-A), circumpapillary RNFL thickness (cpRNFT), pattern standard deviation (PSD), mean deviation (MD), and threshold of sensitivity (TS) (x-axis) was generated to calculate a correlation coefficient. Only data with significant correlations are shown. r = correlation coefficient from the fitted linear regression model.

Fig 3A–3C shows the correlations between FD, the disappearance angle of the RPCs, and other parameters. Fig 4 shows scatterplots illustrating the linear associations between flow density on OCT angiography, the disappearance angle of the RPCs, and other factors in glaucoma. FD was significantly correlated with the disappearance angle of the RPCs (r = -0.448, P = 0.001), the angle of the RNFL defect (r = -0.445, P = 0.001), cpRNFL thickness (r = 0.715, P<0.001), PSD value (r = 0.471, P<0.001), MD value (r = 0.558, P<0.001), sensitivity threshold (r = 0.503, P<0.001), and age (r = -0.303, P<0.05). The disappearance angle of the RPCs was significantly correlated with the angle of the RNFL defect (r = 0.966, P<0.001), cpRNFL thickness (r = -0.496, P<0.001), PSD value (r = 0.288, P<0.05), MD value (r = -0.508, P<0.001), and sensitivity threshold (r = -0.532, P<0.001). The angle of the RNFL defect was correlated with cpRNFL thickness (r = -0.453, P = 0.001), PSD value (r = 0.353, P<0.05), MD value (r = -0.539, P<0.001), and sensitivity threshold (r = -0.556, P<0.001). cpRNFL thickness was correlated with PSD value (r = -0.56, P<0.001), MD value (r = 0.706, P<0.001), sensitivity threshold (r = 0.641, P<0.001), and age (r = -0.323, P<0.05), and MD value was correlated with sensitivity threshold (r = 0.936, P<0.001). Age was correlated with MD value (r = -0.379, P<0.01) and sensitivity threshold (r = -0.419, P<0.01) in the overall population (Fig 3A). FD was significantly correlated with the disappearance angle of the RPCs (r = -0.707, P<0.001), the angle of the RNFL defect (r = -0.68, P<0.001), cpRNFL thickness (r = 0.814, P<0.001), and PSD value (r = -0.445, P<0.05). The disappearance angle of the RPCs was significantly correlated with the angle of the RNFL defect (r = 0.991, P<0.001), cpRNFL thickness (r = -0.677, P = 0.001), MD value (r = -0.559, P = 0.01), and sensitivity threshold (r = -0.586, P<0.01). The angle of the RNFL defect was correlated with cpRNFL thickness (r = -0.633, P<0.01), MD value (r = -0.571, P<0.01), and sensitivity threshold (r = -0.568, P<0.01). cpRNFL thickness was correlated with sensitivity threshold (r = 0.493, P<0.05) and SE (r = -0.513, P<0.05). MD value was correlated with sensitivity threshold (r = 0.917, P<0.001) and age (r = -0.756, P<0.001). Sensitivity threshold was correlated with age (r = -0.752, P<0.001) in the POAG patients (Fig 3B). FD was significantly correlated with the disappearance angle of the RPCs (r = -0.362, P<0.05), the angle of the RNFL defect (r = -0.391, P<0.05), cpRNFL thickness (r = -0.616, P<0.001), PSD value (r = -0.464, P<0.01), MD value (r = 0.524, P<0.01), and sensitivity threshold (r = 0.553, P = 0.001). The disappearance angle of the RPCs was significantly correlated with the angle of the RNFL defect (r = 0.955, P<0.001), cpRNFL thickness (r = -0.472, P<0.01), MD value (r = -0.553, P = 0.001), and sensitivity threshold (r = -0.498, P<0.01). The angle of the RNFL defect was correlated with cpRNFL thickness (r = -0.448, P = 0.01), MD value (r = -0.564, P = 0.001), and sensitivity threshold (r = -0.518, P<0.01). In the NTG patients, cpRNFL thickness was correlated with PSD value (r = -0.603, P<0.001), MD value (r = -0.739, P<0.001), and sensitivity threshold (r = 0.746, P<0.001), and MD value was correlated with sensitivity threshold (r = 0.979, P<0.001) and age (r = -0.232, P<0.05) (Fig 3C).

**Fig 5:**
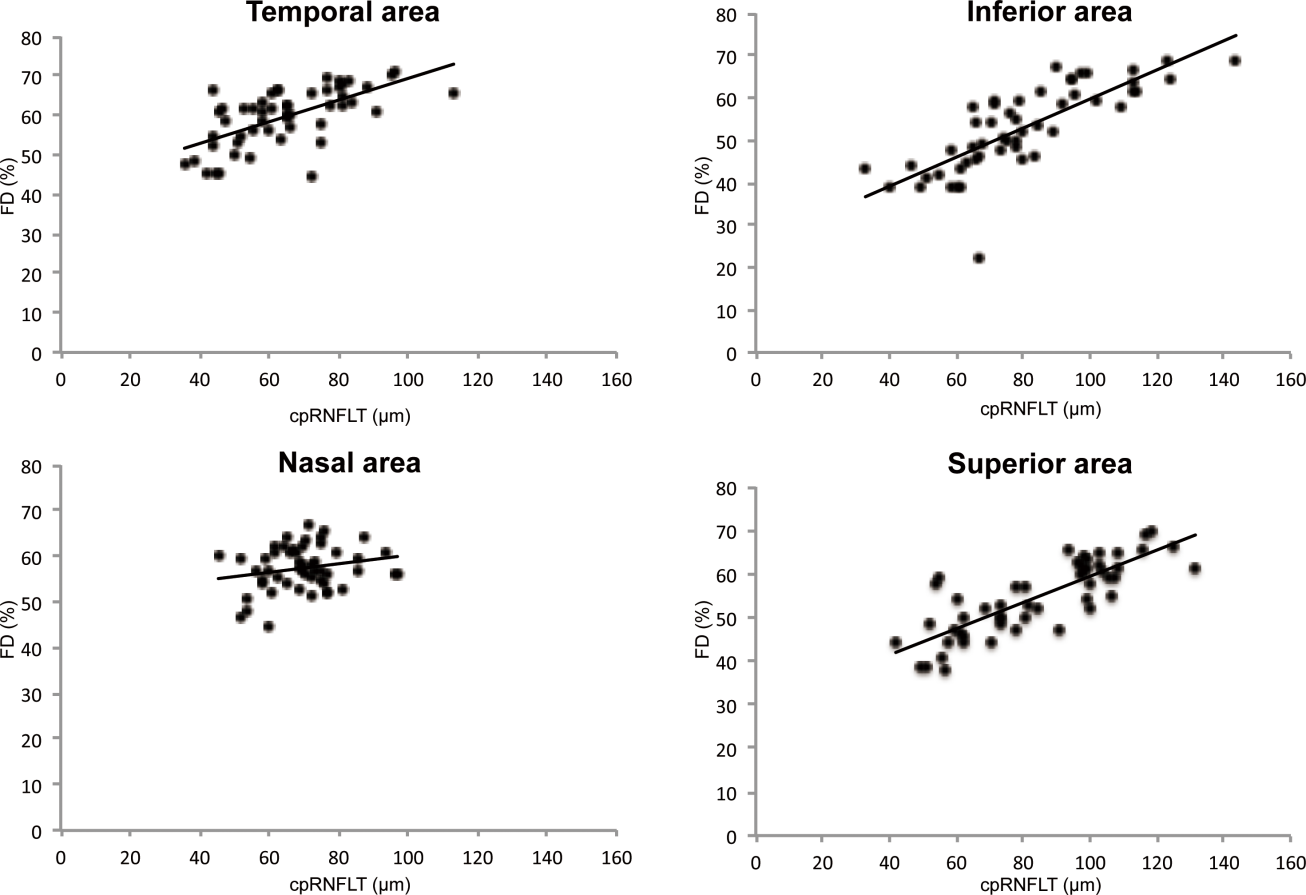
Correlation between flow density on OCT angiography and cpRNFL thickness in different peripapillary segments. The correlation between FD and cpRNFL thickness was analyzed in the four segments generated in Fig 2, and the actual measurement data are shown in Table 3. A scatter plot of FD (y-axis) and cpRNFL thickness (x-axis) was generated to calculate a correlation coefficient. r = correlation coefficient from the fitted linear regression model.

The correlation between FD and cpRNFL thickness varied by peripapillary segment (Table 3, Fig 5). In the overall study population, significant correlations were observed in the temporal segment (r = -0.653, P<0.001), superior segment (r = 0.803, P<0.001), and inferior segment (r = 0.851, P<0.001), but not in the nasal segment (r = 0.139, P = 0.327). In the POAG patients, significant correlations were observed in the temporal segment (r = 0.645, P<0.001), superior segment (r = 0.795, P<0.001), and inferior segment (r = 0.817, P<0.001), but not in the nasal segment (r = 0.233, P = 0.346). In the NTG patients, significant correlations were observed in the temporal segment (r = 0.654, P<0.001), superior segment (r = 0.727, P<0.001), and inferior segment (r = 0.876, P<0.001), but not in the nasal segment (r = 0.58, P = 0.751).

**Table 3:** The correlation between flow density on OCT angiography and cpRNFL thickness varied by peripapillary segment.

		cpRNFLT of OCT analysis of optic disc (μm)	Flow density of OCT Angiography (%)	r value (correlation coefficient)	P value
	
Temporal area	Total	60,70±16.83	59.72±7.23	0.653	**0.001**
	POAG	57.02±15.21	57.57±7.32	0.645	**0.002**
	NTG	69.49±1621	60.90±6.91	0.654	**<0.001**
Superior area	Total	84.18±22.69	54.74±8.42	0.803	**<0.001**
	POAG	80.70±23.36	52.17±8.01	0.795	**<0.001**
	NTG	86.35±22.36	56.19±8.25	0.727	**<0.001**
Nasal area	Total	69.74±11.11	57.44±4.81	0.139	0.327
	POAG	70.13±11.83	56.65±4.95	0.223	0.346
	NTG	69.50±10.83	57.96±4.74	0.58	0.751
Inferior area	Total	79.68±22.91	52.83±10.01	0.851	**<0.001**
	POAG	71.67±17.15	51.58±8.72	0.817	**<0.001**
	NTG	84.69±24.82	53.57±10.76	0.876	**<0.001**

Statistically significant correlations (P<0.01) are indicated in bold.

## Discussion

When fluorescein angiography was the only modality capable of measuring intraocular pressure, it was recommended that while undergoing this procedure, patients with normal-tension glaucoma should be checked for any delay in blood flow and that intraocular pressure should be measured [6]. This precaution was due to the fact that in addition to intraocular pressure, changes in hemodynamics are thought to contribute to glaucoma, supporting the significance of measuring blood flow in and around the optic disc in patients with glaucoma. In recent years, the measurement of ocular blood flow has become easier and non-invasive owing to the development of LSFG and OCT angiography. In line with the principle of SSADA, OCT angiography measures blood flow by capturing any objects moving inside the retina as blood cells in the vessels. In other words, OCT angiography simply observes the shapes of blood vessels where blood cells travel, instead of detecting blood vessels, capillary networks, or blood flow itself. In addition, OCT angiography is safe and non-invasive because it enables the observation of the intraocular capillary networks within a few seconds of visual fixation, offering a tremendous advantage. While fluorescein angiography images provide a panoramic, stereoscopic view of vascular networks inside the eyes, OCT angiography enables the observation of individual retinal layers according to the level of segmentation. Furthermore, recent studies have used OCT angiography for quantitative assessments of vascular density and blood flow [12,13], suggesting that this modality has wide clinical applications.

Many studies have already used OCT angiography to examine the optic discs of patients with glaucoma. Using the SSADA algorithm, Jia et al. performed non-invasive OCT angiography to stereoscopically examine the intrapapillary vasculature in patients with glaucoma; this technique semi-automatically quantified optic disc perfusion, revealing a significant decline in the optic disc flow index in glaucoma [12]. They reported that the reproducibility of SSADA-based OTC angiography was far superior to that of laser Doppler flowmetry. In a subsequent study of glaucoma, the same research group showed that OCT angiography was capable of displaying a reduction in blood perfusion in the peripapillary retina as a local defect, or quantified as a peripapillary flow index or blood vessel density. The peripapillary flow index was an especially good indicator of the severity of glaucoma compared with RNFL thickness [13]. Wang et al. also performed OCT angiography in patients with glaucoma and showed a strong correlation between ganglion cell complex thickness and reductions in flow index and vessel density [14], suggesting that it is important to measure blood flow in the optic disc during follow-up observation. Furthermore, a study performing speckle dispersion OCT angiography in healthy individuals, patients with glaucoma, and people suspected of having glaucoma reported a strong correlation between the density of RPC and RNFL thickness or visual field index [15]. Thus, many studies have shown a correlation between OCT angiography findings, vessel density, and peripapillary blood flow in and around the optic disc in patients with glaucoma [16–24].

This study also demonstrated that the disappearance angle of the RPC was significantly correlated with three functional indicators: PSD values, MD values, and sensitivity threshold. The disappearance angle of the RPC may be a useful morphological change to reference when evaluating functional changes related to glaucomatous optic neuropathy. A correlation between the disappearance angle of the RPC and the angle of the RNFL defect, a morphological indicator, again suggests that the former is a morphological change associated with glaucomatous optic neuropathy. These findings suggest that the progression of glaucoma may be predicted by the disappearance angle of the RPC.

In this analysis, patients in the POAG group had lower MD, DA, and NFLD-A than those in the NTG group. These results show that POAG and NTG are not identical pathological conditions. As previously reported, patients with POAG show more diffuse visual field damage than those with NTG [25].

Similarly, in this study, FD was significantly correlated with functional indicators such as PSD values, MD values, and sensitivity threshold. FD was also significantly correlated with cpRNFL thickness, a morphological indicator, and the strongest correlation occurred in the inferior segment followed by the superior and then temporal segments. These correlations suggest that the superior and inferior regions of the optic disc are affected more strongly by glaucoma, which is a pathological feature of glaucomatous optic neuropathy. Age was the only clinical characteristic showing a significant correlation (<5% level) in this study. In a study of LSFG in 107 healthy eyes, Tamura et al. reported a negative correlation between age and blowout score, the latter reflecting ease of blood flow, suggesting that attention must be paid to patient age when investigating changes in FD [26]. After careful consideration of these notable aspects of the clinical background, it can be considered that FD may be a useful indicator of functional and morphological changes in glaucomatous optic neuropathy.

In this study, we performed OCT angiography to evaluate the intrapapillary and peripapillary vascular networks in patients with glaucoma, and compared quantitative OCT angiography indicators, such as flow index and vessel density, SD-OCT indicators such as peripapillary RNFL thickness, and visual field indicators. However, further study is needed because of possible variations in actual blood flow and the potential influence of systemic complications, eye drops, and oral medications. Our previous and present studies enrolled relatively small numbers of patients, necessitating a large-scale, long-term study in the future. In addition, the present intrapapillary observation method tends to generate segmentation errors, and the cross-section for observation is fixed according to the distance from the surface of the optic disc. In other words, the tissues and layers in each cross-sectional image may differ between the thick prelaminar region in healthy individuals and the extremely thin prelaminar region in patients with glaucoma. Therefore, the establishment of standard observation and measurement methods is needed to verify the comparisons between patients and studies.

## References

[1] Ojima T, Tanabe T, Hangai M, Yu S, Morishita S, Yoshimura N. Measurement of retinal nerve fiber layer thickness and macular volume for glaucoma detection using optical coherence tomography. Jpn J Ophthalmol. 2007;51(3):197–203. doi: 10.1007/s10384-006-0433-y 17554482 10.1007/s10384-006-0433-y

[2] Yamashita T, Ieki Y, Goto K, Kiryu J, Kaji K, Tabuchi A. [A correlation between visual impairment and the thickness of the retinal nerve fiber layer and of the inner retinal layers at the macula measured using spectral-domain OCT]. Atarashii Ganka. 2009;26(7):997–1001.

[3] [The Japan Glaucoma Society Guidelines for Glaucoma (3rd Edition)]. Nippon Ganka Gakkai Zasshi. 2012;116(1):3–46. 22352070

[4] Srinivasan VJ, Radhakrishnan H, Lo EH, Mandeville ET, Jiang JY, Barry S, et al OCT methods for capillary velocimetry. Biomed Opt Express. 2012;3(3):612–629. doi: 10.1364/BOE.3.000612 22435106 10.1364/BOE.3.000612 PMC3296546

[5] Jia Y, Tan O, Tokayer J, Potsaid B, Wang Y, Liu JJ, et al Split-spectrum amplitude-decorrelation angiography with optical coherence tomography. Opt Express. 2012;20(4):4710–4725. doi: 10.1364/OE.20.004710 22418228 10.1364/OE.20.004710 PMC3381646

[6] Spaeth GL: The pathogenesis of nerve damage in glaucoma. Grune & Stratton, New York; 1977. pp. 79–89.

[7] Mo S, Phillips E, Krawitz BD, Garg R, Salim S, Geyman LS, et al Visualization of Radial Peripapillary Capillaries Using Optical Coherence Tomography Angiography: The Effect of Image Averaging. PLoS One. 2017; 12(1):e0169385 doi: 10.1371/journal.pone.0169385. eCollection 2017. 28068370 10.1371/journal.pone.0169385 PMC5222511

[8] Iwata K. Three-dimensional fluoroscopy for evaluation of optic disc vascular network in patients with glaucoma. Therapeutic Research 11:3369–3372, 1990.

[9] Yamazaki S, Inoue Y, Yoshikawa K. Peripapillary fluorescein angiographic findings in primary open angle glaucoma. Br J Ophthalmol. 1996;80(9):812–817. PMCID: PMC505618 8942378 10.1136/bjo.80.9.812 PMC505618

[10] Sugiyama T, Shibata M, Kojima S, Ueki M, Ikeda T. [Stereoscopic fluoroscopic evaluation of changes in blood flow waveforms in the optic disc of patients with glaucoma: LSFG-NAVITM analysis]. Atarashii Ganka. 2012;29(7): 984–987.

[11] Anderson DR, Patella VM: Automated static perimetry. 2nd ed. Mosby. St. Louis; 1999. pp. 121–190.

[12] Jia Y, Wei E, Wang X, Zhang X, Morrison JC, Parikh M, et al Optical tomography angiography of optic disc perfusion in glaucoma. Ophthalmology. 2014;121(7): 1322–1332. doi: 10.1016/j.ophtha.2014.01.021 PMCID: PMC4082728 24629312 10.1016/j.ophtha.2014.01.021 PMC4082728

[13] Liu L. Jia Y. Takusagawa HL, Pechauer AD, Edmunds B, et al Optical tomography angiography of the peripapillary retina in glaucoma. JAMA Ophthalmol. 2015;133(9)1045–1052. doi: 10.1001/jamaophthalmol.2015.2225 PMCID: PMC4950955 26203793 10.1001/jamaophthalmol.2015.2225 PMC4950955

[14] Wang X, Jiang C, Ko T, Kong X, Yu X, Min W, et al Correlation between optic disc perfusion and glaucomatous severity in patients with open-angle glaucoma: an optical coherence tomography angiography study. Graefes Arch Clin Exp Ophthalmol. 2015; 253(9):1557–1564. doi: 10.1007/s00417-015-3095-y 26255817 10.1007/s00417-015-3095-y

[15] Yarmohammadi A, Zangwill LM, Diniz-Filho A, Suh MH, Manalastas PI, Fatehee N, et al Optical Coherence Tomography Angiography Vessel Density in Healthy, Glaucoma Suspect, and Glaucoma Eyes. Invest Ophthalmol Vis Sci. 2016; 57(9): OCT451–OCT459. doi: 10.1167/iovs.15-18944 PMCID: PMC4968912 27409505 10.1167/iovs.15-18944 PMC4968912

[16] Holló G. Vessel density calculated from OCT angiography in 3 peripapillary sectors in normal, ocular hypertensive, and glaucoma eyes. Eur J Ophthalmol. 2016; 26(3):e42–45. doi: 10.5301/ejo.5000717 26692060 10.5301/ejo.5000717

[17] Lévêque PM, Zéboulon P, Brasnu E, Baudouin C, Labbé A. Optic Disc Vascularization in Glaucoma: Value of Spectral-Domain Optical Coherence Tomography Angiography Optic Disc Vascularization in Glaucoma: Value of Spectral-Domain Optical Coherence Tomography Angiography. J Ophthalmol. 2016; 2016:6956717 doi: 10.1155/2016/6956717 26998352 10.1155/2016/6956717 PMC4779818

[18] Bojikian KD, Chen CL, Wen JC, Zhang Q, Xin C, Gupta D, et al Optic Disc Perfusion in Primary Open Angle and Normal Tension Glaucoma Eyes Using Optical Coherence Tomography-Based Microangiography. PLoS One. 2016; 11(5): e0154691 doi: 10.1371/journal.pone.0154691 27149261 10.1371/journal.pone.0154691 PMC4858256

[19] Chen CL, Bojikian KD, Gupta D, Wen JC, Zhang Q, Xin C, et al Optic nerve head perfusion in normal eyes and eyes with glaucoma using optical coherence tomography-based microangiography. Quant Imaging Med Surg. 2016; 6(2):125–133. doi: 10.21037/qims.2016.03.05 27190764 10.21037/qims.2016.03.05 PMC4858460

[20] Mammo Z, Heisler M, Balaratnasingam C, Lee S, Yu DY, Mackenzie P, et al Quantitative Optical Coherence Tomography Angiography of Radial Peripapillary Capillaries in Glaucoma, Glaucoma Suspect and Normal Eyes. Am J Ophthalmol. 2016;170:41–49. doi: 10.1016/j.ajo.2016.07.015 27470061 10.1016/j.ajo.2016.07.015

[21] Lee EJ, Lee KM, Lee SH, Kim TW. OCT Angiography of the Peripapillary Retina in Primary Open-Angle Glaucoma. Invest Ophthalmol Vis Sci. 2016; 57(14):6265–6270 doi: 10.1167/iovs.16-20287 27849312 10.1167/iovs.16-20287

[22] Scripsema NK, Garcia PM, Bavier RD, Chui TY, Krawitz BD, Mo S, et al Optical Coherence Tomography Angiography Analysis of Perfused Peripapillary Capillaries in Primary Open-Angle Glaucoma and Normal-Tension Glaucoma. Invest Ophthalmol Vis Sci. 2016; 57(9): OCT611–OCT620. doi: 10.1167/iovs.15-18945 27742922 10.1167/iovs.15-18945

[23] Akagi T, Iida Y, Nakanishi H, Terada N, Morooka S, Yamada H, et al Microvascular Density in Glaucomatous Eyes with Hemifield Visual Field Defects: An Optical Coherence Tomography Angiography Study. Am J Ophthalmol. 2016; 168:237–249. doi: 10.1016/j.ajo.2016.06.009 27296492 10.1016/j.ajo.2016.06.009

[24] Yarmohammadi A, Zangwill LM, Diniz-Filho A, Saunders LJ, Suh MH, Wu Z, et al Peripapillary and Macular Vessel Density in Patients with Glaucoma and Single-Hemifield Visual Field Defect. Ophthalmology. 2017; 124(5):709–719. doi: 10.1016/j.ophtha.2017.01.004. Epub 2017 Feb 10. 28196732 10.1016/j.ophtha.2017.01.004 PMC5499385

[25] Fukuchi T, Yoshino T, Sawada S, Seki M, Togano T, Tanaka T, et al Progression rate of total, and upper and lower visual field defects in open-angle glaucoma patients. Clin Ophthalmol. 2010; 18(4):1315–23. doi: 10.2147/OPTH.S13101 10.2147/OPTH.S13101 PMC2993106 21139672

[26] Tamura A, Kogure A, Watanabe G, Kishi G, Hori S. [Association between age and chorioretinal hemodynamics in normal volunteers examined with laser speckle flowgraphy]. Jpn J Ophthalmol. 2013; 117(2):110–116. . 23534255

